# Supplementary material for: The interactome of intact mitochondria by cross-linking mass spectrometry provides evidence for coexisting respiratory supercomplexes
Source: Mol Cell Proteomics. 2017 Dec 8;17(2):216–32. doi: 10.1074/mcp.RA117.000470 (PMC5795388; doi:10.1074/mcp.RA117.000470)
Supplement: Supplemental Data [file supp_RA117.000470_133922_0_supp_23978_fzffwf.zip › spectra_annotation/mito_DR_spectra_annotation/372-1-11-1-6-1.pdf]

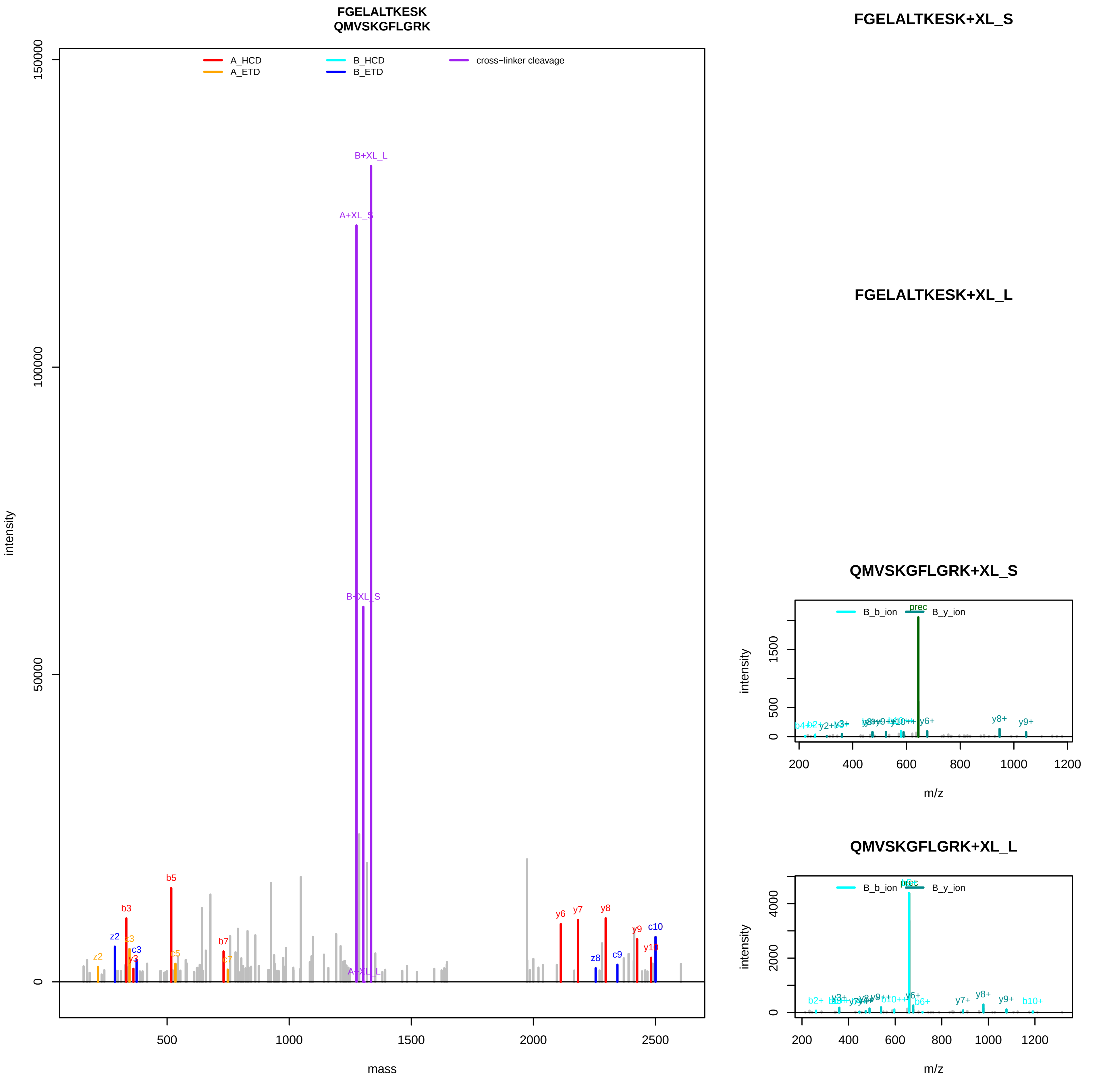

FGELALTKESK
QMVSKGFLGRK
A_HCD
A_ETD
B_HCD
B_ETD
cross−linker cleavage
B+XL_L
A+XL_S
B+XL_S
A+XL_L
b3
b5
b7
z2
z3
c3
y3
c5
c7
y6
y7
y8
y9
y10
z8
c9
c10
intensity
mass
0
50000
100000
150000
500
1000
1500
2000
2500
FGELALTKESK+XL_S
FGELALTKESK+XL_L
QMVSKGFLGRK+XL_S
QMVSKGFLGRK+XL_L
B_b_ion
B_y_ion
prec
y6+
y8+
y9+
b2+
y7+
b6+
b10+
m/z
intensity
0
500
1500
2000
4000
200
400
600
800
1000
1200
